# Is there any benefit to adding students to the European council on chiropractic education evaluation teams and general council? An audit of stakeholders

**DOI:** 10.1186/s12998-019-0274-7

**Published:** 2019-10-13

**Authors:** Cynthia Peterson, Joyce Miller, B. Kim Humphreys, Ken Vall

**Affiliations:** 1 European Council on Chiropractic Education, Aachen, Germany; 2 Department of Chiropractic, Faculty of Health, University of Johannesburg, Johannesburg, South Africa; 3 Ango-European College of Chiropractic, University College, 13-15 Parkwood Road, Bournemouth, England; 4 Faculty of Medicine, University of Zurich, Zurich, Switzerland

**Keywords:** Chiropractic, Education, Accreditation, Students

## Abstract

**Background:**

The European Council on Chiropractic Education (ECCE) is currently the only chiropractic specific accrediting body in the world to include students as equal members on Council and accreditation evaluation teams. Therefore, the purpose of this study is to evaluate feedback from four ECCE stakeholder groups regarding the effectiveness of chiropractic students on ECCE General Council and evaluation teams.

**Methods:**

This was a mixed-methods audit using questionnaires including closed statements requesting level of agreement and open-ended statements requesting written responses. The proportion of responses falling into the five categorical options for level of agreement was calculated for each questionnaire using descriptive statistics. The analysis of the two statements per questionnaire requiring written responses used a modified ‘thematic analysis’ approach. Three researchers independently identified themes from the written responses. They then met to agree the final themes for each statement.

**Results:**

The response rates for the four questionnaires ranged from 87 to 100%. Feedback regarding ‘Student members on General Council’ was the least positive with 65% neutral or negative regarding ‘students being prepared for meetings’. Feedback from stakeholders regarding use of students on evaluation teams was universally positive, ranging from 82.4–100% Strongly Agreeing or Agreeing with each closed statement.

Themes were identified for each open statement. The unique contribution students make to evaluation teams was most common. General Council feedback identified ‘lack of student preparation’ and ‘the short time period of student membership’ as important themes.

**Conclusions:**

This study demonstrates the unique and positive contributions chiropractic students make to accreditation evaluation teams. The results were less positive concerning students on ECCE General Council due to the lack of specific training for their roles and the short time-frame of their membership. Therefore, the ECCE has created training workshops and expanded the time period for students on Council in order to address these issues.

**Electronic supplementary material:**

The online version of this article (10.1186/s12998-019-0274-7) contains supplementary material, which is available to authorized users.

## Introduction

The European Council on Chiropractic Education (ECCE) is an autonomous, international organization whose primary focus is on accreditation (and reaccreditation) of institutions offering chiropractic education and training [1]. Accreditation (and reaccreditation) of institutions is determined by the quality of their chiropractic education and training programmes judged against a set of educational Standards. These Standards, which themselves are evaluated and updated regularly, are intended for use by chiropractic institutions, in both the private and public sectors for self-evaluation of their educational programmes as well as for use by international bodies involved in the recognition and accreditation of chiropractic education worldwide [1]. Currently, the ECCE evaluates 10 programmes in Europe and South Africa.

The ECCE was started by the European Chiropractors’ Union (ECU) in 1981, but has only included two students on General Council and one student on each accreditation evaluation team since 2011 [1–4]. The ECCE is the only chiropractic specific accreditation body in the world that includes student members [2–4]. The two students on General Council are nominated by the student bodies of the accredited institutions but serve only 1 or 2 years on Council prior to their graduation [5]. The student members of each accreditation evaluation team are selected by the ECCE’s Executive committee based on recommendations from the management of one of the accredited institutions not involved in the current evaluation. Student members of evaluation teams serve only once due to the fact they are normally final year students at the time of the evaluation event.

It has been generally accepted that students provide unique and positive perspectives to ECCE’s activities and thus are important stakeholders. However, no data have been collected to assess these assumptions based on experiences of ECCE General Council members, non-student accreditation evaluation team members, accredited institutions/programs and the involved students themselves. In particular, it seems important to assess perceptions of students’ usefulness and specific contributions, if any, to ECCE’s activities. There are real and logical benefits for the ECCE to understand the role and effectiveness of student members, not least because there are cost implications. Additionally, the ECCE needs to identify if there are areas of concern regarding the effectiveness of students on council and evaluation teams so that changes in ECCE policies and procedures can occur to remedy deficiencies. Therefore, the aim of this study was to collect feedback from four ECCE stakeholder groups regarding their perceptions and implications of including chiropractic students on the ECCE General Council and accreditation evaluation teams.

## Methods

This was a mixed-methods audit using questionnaires which included both closed statements requesting a level of agreement, thus providing percentage agreement for each statement option, and open-ended questions/statements requesting written responses. The written responses were particularly important to identify specific issues that may be associated with those statements having a higher percentage of less positive responses.

Four similar questionnaires were designed, one for each of the four stakeholder groups (General Council, Accredited Institutions, Evaluation Team Members who were not students, and Student Evaluation Team Members) (Additional files 1, 2, 3 and 4). The ECCE Quality Assurance Consultant, who had been a member of ECCE for 8 years and who had also participated on several evaluation teams, created the first drafts of each questionnaire. The specific questions selected for inclusion arose from feedback questionnaires used by ECCE for past evaluation visits and modified for use by General Council. The questionnaires were then sent to the ECCE Quality Assurance committee for peer review and revision. Minor revisions of wording were agreed. Finally, the questionnaires were sent to the ECCE executive committee for input, revision and agreement of the final versions. No further recommendations for questionnaire revision arose. Two of the questionnaires (non-student Evaluation Team Member; Accredited Institution) included six closed statements, each requesting the respondents to identify their level of agreement to each statement using the five options: Strongly Agree; Agree; Neither Agree nor Disagree; Disagree; Strongly Disagree (Additional files 2 and 3). The General Council stakeholder group’s questionnaire and the Student Evaluation Team member questionnaire each had five closed statements using the same options (Additional files 1 and 4).

Each questionnaire also included two open-ended statements/questions requesting written responses. There was no limit to the length of any response. These four ‘Additional files’ show the complete questionnaires. Three of the questionnaires asked for ‘Positive comments about your experiences having students on Council or Evaluation Teams’ as well as ‘Areas for improvement needed based on your experiences having students on Council or Evaluation Teams.’ For the questionnaire completed by previous students on evaluation teams, the open-ended questions were: 1) Overall, the strong points of my experiences as a student member of the evaluation team were: 2) What unique contributions did you, as a student, make to the team and the evaluation process?

Questionnaires to General Council members were hard copies, completed individually and collected by the Quality Assurance consultant immediately prior to the start of the November 2018 Council meeting held in London, England. The first author of this study was in attendance and provided the instructions to all in attendance and monitored that no discussions occurred while completing the questionnaires. No identifying personal information was included on any of the questionnaires. Similarly, hard copy questionnaires from the Principals/Department Heads of nine of the ten accredited Institutions were completed independently prior to the start of the November 2018 meetings. One institution was not in attendance so that particular questionnaire was then sent electronically to the Department Head and returned via email. As a result, this particular questionnaire was not anonymous. Questionnaires to the non-student evaluation team members in attendance at the General Council meeting were also completed independently on hard copies and collected not only at the November 2018 Council meeting to those participants who had also been on previous evaluation teams but also sent electronically via email to prior evaluation team members not on ECCE General Council. All questionnaires to students were sent electronically and collected via email. Therefore, the responses collected electronically were not necessarily completely anonymous, but names were not added to the printed and saved questionnaires returned. For those failing to respond to the first email request, a second email reminder was sent. For all stakeholder groups, completion of the questionnaire was considered informed consent to participate in the study.

### Data analysis

#### Categorical responses

The proportion of overall closed statement responses falling into each of the five categorical options for each statement (strongly agree, agree, neither agree nor disagree, disagree, strongly disagree) was calculated for the four questionnaires using descriptive statistics. Percentage agreements for each category were calculated.

#### Written responses

The analysis of the two questions/statements on each questionnaire requiring written responses used a modified ‘thematic analysis’ approach [6]. All responses to the two written questions/statements for each questionnaire were first copied ‘verbatum’ into a single table (see Additional file 5) and distributed to three researchers. Each researcher independently studied and evaluated all responses to each statement/question with the instructions to identify recurrent ‘themes’ from the responses for each written statement/question. Once each of the three researchers completed their independent evaluations of the written responses, they met to discuss their findings and agree the final ‘themes’ for responses to each statement/question. The process of agreeing the final themes started with the initial ‘themes’ independently identified by each of the three researchers being written onto a large paper covered easel by one of the researchers in order to visualize these initial thematic ideas and to facilitate discussion. Each of the three researchers then explained how and why they had arrived at their particular categories. Discussion then followed. There were no serious disagreements between the three researchers regarding the final themes with most of the discussions focused on the precise wording for each mutually agreed theme.

Ethical approval was not necessary for this audit study with voluntary participation and no interventions. Returning the completed questionnaire was considered informed consent to participate and participants were informed of this.

## Results

The response rates for the four different questionnaires were: 100% for both the Institutional feedback (10 responses) and Students on Evaluation Teams feedback (12 responses) questionnaires, 87.0% for the General Council members (20/23 responses) and 89.5% (17/19 responses) for the Evaluation Team members who were not students.

### Categorical data: closed statement findings

Table 1 shows the number of respondents regarding the use of students on ECCE General Council (*n* = 20) who ‘strongly agree’, ‘agree’, ‘neither agree nor disagree’, ‘disagree’, or ‘strongly disagree’ for each of the five closed statements. A high proportion of positive responses (strongly agree or agree) were found for two of the five statements. These were ‘Students are treated as equal members of ECCE council’ (95%) and ‘Student members of ECCE council behave professionally at all times’ (100%). The closed statement with the highest proportion of Council members responding negatively or neither agreeing nor disagreeing with the statement was ‘Student members are well prepared for ECCE Council meetings’ with 65% providing neutral or negative responses (Table 1). Additionally, 35% of respondents were neutral, disagreed or strongly disagreed with the statement ‘Students make unique contributions to the ECCE General Council in terms of student needs and perspectives’ and 30% of council members were neutral or disagreed with the statement ‘Student members of ECCE council ask appropriate questions during meetings’ (Table 1).

**Table 1:** General council feedback on the use of students on ECCE general council (20 out of maximum 23 completed questionnaires (87%))

	Strongly agree	Agree	Neither agree nor disagree	Disagree	Strongly disagree
Students are treated as equal members of ECCE General Council	10/20 (50%)	9/20 (45%)	1/20 (5%)	0/20	0/20
Student members are well prepared for ECCE Council meetings	1/20 (5%)	6/20 (30%)	8/20 (40%)	3/20 (15%)	2/20 (10%)
Student members of ECCE council behave professionally at all times.	9/20 (45%)	11/20 (55%)	0/20	0/20	0/20
Student members of ECCE council ask appropriate questions during meetings.	3/20 (15%)	11/20 (55%)	5/20 (25%)	0/20	1/20 (5%)
Students make unique contributions to the ECCE General Council in terms of student needs and perspectives.	4/20 (20%)	9/20 (45%)	4/20 (20%)	2/20 (10%)	1/20 (5%)

Table 2 shows the categorial distribution of responses submitted by the non-student Evaluation Team members (*n* = 17) for each of the six closed statements. All of the respondents (100%) agreed or strongly agreed that the student team members were a) treated as equals to the other team members and b) behaved professionally at all times. The majority of evaluation team members also agreed or strongly agreed that the student members asked appropriate questions during meetings (88.2%), made unique contributions to the evaluation team (94.1%), and were treated as equal to the non-student members by the institution (88.2%). Most non-student evaluation team members agreed or strongly agreed that the student team members were well prepared for the evaluation visit (82.4%), but one responder disagreed with this statement.

**Table 2:** Evaluation team feedback (non-Student members) regarding the use of students on ECCE evaluation teams (17 out of 19 maximum completed questionnaires returned (89.5%))

	Strongly agree	Agree	Neither agree nor disagree	Disagree	Strongly disagree
Students were treated as equal members of ECCE Evaluation teams	10/17 (59%)	7/17 (41%)	0/17	0/17	0/17
Student members were well prepared for the evaluation	3/17 (18%)	11/17 (65%)	2/17 (12%)	1/17 (6%)	0/17
Student members of the evaluation team behaved professionally at all times.	14/17 (82%)	3/17 (18%)	0/17	0/17	0/17
Student members of evaluation teams asked appropriate questions during meetings with institutional representatives.	9/17 (53%)	6/17 (35%)	2/17 (12%)	0/17	0/17
Student members made unique contributions to the Evaluation team.	8/17 (47%)	8/17 (47%)	1/17 (6%)	0/17	0/17
Student members were treated as equal to the non-student members by the institution.	10/17 (59%)	5/17 (29%)	2/17 (12%)	0/17	0/17

Feedback from the ten accredited chiropractic programs/institutions to the six closed statements is shown in Table 3. Four of the six closed statements had no recorded disagreement. Of the remaining two closed statements, one institution disagreed that ‘Student members of Evaluation teams asked appropriate questions during meetings’ and ‘Student team members were treated as equal to the non-student team members by individuals in your institution.’ All respondents (100%) strongly agreed or agreed that the student evaluation team members were treated as equal to the non-student members by the other members of the evaluation team, however. All but one institution (90%) agreed or strongly agreed that the student members of the evaluation teams made unique contributions to the team. Additionally, 90% of the institutions stated that the student evaluation team members behaved professionally at all times.

**Table 3:** Accredited Institution feedback on the use of students on ECCE evaluation teams. (100% of possible questionnaires returned. *N* = 10)

	Strongly agree	Agree	Neither agree nor disagree	Disagree	Strongly disagree
Students were treated as equal members of Evaluation teams	5/10 (50%)	5/10 (50%)	0/10	0/10	0/10
Student members were well prepared for the evaluation event.	1/10 (10%)	7/10 (70%)	2/10 (20%)	0/10	0/10
Student members of Evaluation teams behaved professionally at all times.	8/10 (80%)	1/10 (10%)	1/10 (10%)	0/10	0/10
Student members of Evaluation teams asked appropriate questions during meetings.	4/10 (40%)	3/10 (30%)	2/10 (20%)	1/10 (10%)	0/10
Student members made unique contributions to the Evaluation team.	3/10 (30%)	6/10 (60%)	1/10 (10%)	0/10	0/10
Student team members were treated as equal to the non-student team members by individuals in your institution.	5/10 (50%)	2/10 (20%)	2/10 (20%)	1/10 (10%)	0/10

Table 4 shows the feedback from former student members (*n* = 12) of the evaluation teams. These responses included students from chiropractic programmes in the United Kingdom, Denmark, France, Spain and South Africa. Four of these five questions had 91.7% of the students agreeing or strongly agreeing with each statement. Only the question ‘I felt that as a student member of the evaluation team that I made unique contributions to the evaluation process’ had 75% of respondents agreeing or strongly agreeing, but no student disagreed or strongly disagreed with this statement. Three students were neutral on this issue.

**Table 4:** Student ECCE evaluation team member feedback on their experiences on ECCE evaluation teams (100% of possible questionnaires returned *N* = 12)

	Strongly agree	Agree	Neither agree nor disagree	Disagree	Strongly disagree
As a student member of an evaluation team I was treated as an equal team member by the other non-student members.	8/12 (67%)	3/12 (25%)	1/12 (8%)	0/12	0/12
I was well informed about my duties as a student member of the team prior to the site visit.	9/12 (75%)	2/12 (17%)	1/12 (8%)	0/12	0/12
I felt that as a student member of the evaluation team that I had the opportunity to gather relevant information and contribute to the report in a full and meaningful manner.	10/12 (83%)	1/12 (8%)	0/12	1/12 (8%)	0/12
I felt that as a student member of the evaluation team that I made unique contributions to the evaluation process.	2/12 (17%)	7/12 (58%)	3/12 (25%)	0/12	0/12
I felt that I was treated with respect and as an equal member of the evaluation team by the institution that was being evaluated.	4/12 (33%)	7/12 (58%)	1/12 (8%)	0/12	0/12

Themes Identified from written responses from the four stakeholder groups:

### Statement 1: “Positive comments about your experience of having students on (ECCE General Council, Evaluation Teams)”

I. **ECCE General Council:**

Two themes emerged from the written responses:

1. Importance: Students on General Council are important because they are key stakeholders and as such their perspective has value.
2. Unique perspective: Student perspective is fresh and unparalleled to the rest of the council members.

II. **Evaluation Team Members who were not Students:**

Two themes were identified from the written responses:

1. Unique perspective: students ask questions not approached by other team members.

2. Identification: Better communication with students from the Institution.

III. **Accredited Institutions:**

One theme emerged from the written responses:

Student perspective: The student evaluation team members had a specific focus on the areas unique to students.

### Statement 2: “Areas for improvement needed based on your experience of having students on (ECCE General Council, Evaluation Teams)”

I. **ECCE General Council**

Several council members commented that students did not appear to be prepared for and were unsure of their role on General Council. Two themes arose from the comments to address these problems.

1. Preparation: Much more training of student members is needed prior to their first meeting.
2. Value of experience: Expand the definition of ‘student’ to include those in recognized post-graduate training programs or degrees so that they can remain on General Council for more than 1 or 2 years.

II. **Evaluation Team Members who were not students**

1. Preparation: Better and more timely training: This will empower the student team members by increasing their confidence and help them avoid comparing the institution being accredited with their own institution.
2. Compartmentalize: Assign student members to specific areas of the ‘Standards’ (i.e. those dealing closely with student issues).

III. **Accredited Institutions**

Two themes were identified:

1. Niche: Carefully consider their areas of competence (niching) when assigning their roles.
2. Experience: Use students in the later years of their education or expand the definition of ‘student’ to include those in post-graduate programs.

IV. **Students on Evaluation Teams:**

The written feedback to both open statements on this questionnaire was far and away the most extensive (see Additional file 5).

The themes that arose most frequently to the statement **“Overall the strong points of my experience as a student member of the evaluation team were:”**

1. Personal benefit: A very good experience with a personalized benefit.
2. Student Perspective: I was able to make a unique contribution as I was better able to relate to and understand the students.

Three main themes arose from the question **“What unique contributions did you, as a student, make to the team and the evaluation process?”**

1. Improved communication: The student team members were good communication liaisons between the institution and the ECCE evaluation team (particularly when the student was a native speaker of the language of the institution).
2. Confirmation: Student members of the team can help confirm that the documents reflect the actual experiences, provisions and infra-structure of the institution from the students’ perspective.
3. Insider’s Perspective: Student team members can provide the students’ views of the curriculum.

## Discussion

This survey was the first attempt to gather evidence to determine the value of student involvement in key positions on the ECCE Accreditation teams and General Council through evaluating perceptions of all of the key stakeholders. As such, the preponderance of evidence suggests that the stakeholders found value in the students’ participation. The data reveal a niche where the students excel, along with ideas for improvement. From primarily the written answers from non-student members of evaluation teams and responses to the closed questions, it was clear that student members of the evaluation teams are particularly useful in obtaining information from students at the institution being evaluated during the accreditation evaluation events. Not only did the student team members themselves recognize this, but the institutions and non-student evaluation team members pointed this out as well via their written responses to the open questions. Several respondents pointed out that student evaluation team members were more familiar with student issues and thus better able to formulate appropriate questions to obtain relevant information that non-student evaluation team members could not. Related to this, it was also identified that assigning student members of evaluation teams to explore very specific areas (i.e. ‘Standards’) for their questioning and report write-ups during the evaluation visit would be most effective.

Further, the written feedback from the former student members of evaluation teams was extensive and unanimously positive. Not only did they recognize the unique ways that they contributed to the evaluation visit through their rapport with students from the institution, many provided comments concerning how much they learned themselves from the experience. They also felt respected by the institution and other team members. One of their comments deserves particular attention:

“I had the opportunity to see how this kind of evaluation is made and what are the most important points every college needs to have in order to offer an education of quality. After this experience, I got more involved in the academic part of chiropractic and became a teaching assistant, which has been a great decision for my future as a chiropractor on so many levels. I was able to take advantage of this chance I was given and used it as a way to get out of my comfort zone and get to learn to be less introverted, talk in front of many people in stressful situations. I have always seen this experience as a game changer for me in a very helpful way in which I discovered that I was able to do many things I have never thought I could.”

However, the findings regarding the use of students on the ECCE General Council were not as positive as those for the evaluation teams. The most common problem was that these particular students were not prepared for the General Council meetings, were unsure of their role on council and thus unable to contribute as much as they could. This theme of being unprepared arose almost unanimously amongst the written comments. Following on from this, many council members stated that the student members of General Council needed intensive training prior to their meetings. The ECCE acknowledged that, unlike student members of accreditation evaluation teams, no specific training was offered to student members of General Council prior to their first meeting. In spite of this, most members of the ECCE General Council see the potential of student members and the unique contributions that they could make. Whether or not this difference between the effectiveness of students on the ECCE General Council compared to accreditation evaluation teams is due to the way specific students are selected for these roles is unknown and further research is indicated. Students on the evaluation teams are selected from the management of accredited institutions whereas students on General Council are selected by the student bodies of the accredited institutions. Students selected by the institutional managers for evaluation teams are likely to be the top academic students.

The other main problem with the students on the ECCE General Council, is their very short term of service (i.e. 1–2 years). Several written comments identified this issue. Currently, the students on General Council attend at most two meetings prior to their graduation. This is because students are not selected for General Council membership until they have completed at least 2 years of their chiropractic education so that they have some experience as a chiropractic student. They then graduate at the end of their 4th or 5th year, coming off of council at that time. This is reflected in theme #2 from the ECCE General Council feedback questionnaire on ‘areas for improvement needed’ i.e. “Value of experience: Expand the definition of ‘student’ to include those in recognized post-graduate training programmes or degrees so that they can remain on General Council for more than one or two years.”

In recognition of these two important issues of inadequate preparation and a short term of service arising from this study regarding the use of students on General Council, the ECCE made two important changes. The first is that the ECCE expanded the definition of ‘student’ to include those in recognized post-graduate programs or residencies. Thus, students can remain on General Council beyond 1 or 2 years. The second important change implemented by the ECCE is that the Vice President and the Quality Assurance Consultant have been assigned to create training materials and to conduct training workshops for new student members of council prior to their first meeting. This will address the key finding of this study of lack of preparation. Similar workshops are already in place for evaluation team members.

Although the responses to the three surveys focused solely on the use of students on accreditation evaluation teams, were very positive, one issue stands out where the ECCE can improve. This is to assign the student evaluation team member to specific ‘Standards’ during the accreditation visit and report write-up. This would particularly involve those ‘Standards’ most directly related to the student experiences. This does not mean however, that the student members of the evaluation teams could not be involved in questioning other areas during the evaluation visit.

The findings from this study, showing the specific advantages obtained by including students on chiropractic education accreditation evaluation teams and General Councils may be particularly interesting to the other international chiropractic accrediting bodies that do not currently include students as equal members of their evaluation teams or General Councils [2–4, 7]. They may wish to reconsider their practices based on this evidence. Certainly medical, dental and chiropractic students are integral members on the Swiss Agency of Accreditation and Quality Assurance (AAQ) accreditation evaluation teams for medicine, dentistry and chiropractic in Switzerland, but this does not currently seem to be common practice internationally in medicine [8, 9].

## Conclusions

The results from this study suggest both unique and positive contributions that chiropractic students make to ECCE accreditation evaluation teams. Although the results were slightly less positive concerning students on the ECCE General Council, this is likely due to the lack of specific training for their roles and the short time-frame of their current membership. Based on this study, the ECCE has made important changes to processes and procedures in order to maximize the benefits of student involvement. These include 1) the expansion of our definition of ‘student’ to include those in recognized post-graduate programmes or degrees and 2) creating training materials and workshops for new students prior to membership on council.

### Strengths and limitations to the study

The primary strength of this study was to evaluate the contributions that students make to the ECCE general council and evaluation teams so that improvements can be implemented to maximize their contributions. This is particularly important considering that the ECCE is the only chiropractic specific accrediting body in the world that currently includes students as equal members [2–5] The findings of this study may encourage other chiropractic accrediting bodies to consider including student members.

An additional strength of this study is the very high response rate obtained for the four questionnaires.

The primary limitation of the study would be the questionnaires designed and used to collect the data. Including only 5 or 6 closed questions and 2 open questions may not have captured all of the relevant issues and may have been inherently biased toward specific issues. However, all questionnaires were piloted prior to use and modified based on feedback from ECCE experts. It was desired to use short questionnaires however, to facilitate large response rates.

## Additional files

Additional file 1: The use of students on ECCE General Council. Survey. (PDF 113 kb)

Additional file 2: Evaluation team member feedback on the use of students on ECCE site evaluation teams. Survey. (PDF 120 kb)

Additional file 3: Institution feedback on the use of students on ECCE site evaluation teams. Survey. (PDF 116 kb)

Additional file 4: Student members of ECCE evaluation team: Feedback on the use of students on ECCE evaluation teams. Survey. (PDF 121 kb)

Additional file 5: All Written Comments to Qualitative Questions Regarding the Students on General Council and Evaluation Teams. (PDF 180 kb)

## Data Availability

The datasets generated and analyzed during this study are included in this published article and its supplementary information files.

## Abbreviations

AAQ: Agency for Accreditation and Quality Assurance
ECCE: European Council on Chiropractic Education
