# Supplementary material for: Is there any benefit to adding students to the European council on chiropractic education evaluation teams and general council? An audit of stakeholders
Source: Chiropr Man Therap. 2019 Oct 13;27:53. doi: 10.1186/s12998-019-0274-7 (PMC6790241; doi:10.1186/s12998-019-0274-7)
Supplement: Supplementary file 1 — The use of students on ECCE General Council. Survey. (PDF 113 kb) [file 12998_2019_274_MOESM1_ESM.pdf]

Additional File 1 (PDF): THE USE OF STUDENTS ON ECCE *GENERAL COUNCIL*

The ECCE has had 2 students on the ECCE General Council for the past several years. Students are to be considered as equal members of Council and treated accordingly. After several years of experience using students on ECCE General Council, we would like your feedback on the value and contribution of student members of the ECCE general council.

Please read through each of the questions below and select 1 of the 5 answer options to show your level of agreement with each statement. There are 2 further questions at the end where you can record any comments. Thanks so much for your feedback.

1. The student members of the ECCE General Council are treated as equal Council members by the other non-student members.

Strongly Agree Agree Neither Agree nor Disagree Disagree Strongly Disagree

****

2. Student Council members are well prepared for the ECCE General Council meetings.

Strongly Agree Agree Neither Agree nor Disagree Disagree Strongly Disagree

****

3. Student Council members behave professionally at all times.

Strongly Agree Agree Neither Agree nor Disagree Disagree Strongly Disagree

****

4. Student Council members ask appropriate questions during ECCE Council meetings.

Strongly Agree Agree Neither Agree nor Disagree Disagree Strongly Disagree

****

5. Student members make unique contributions to the ECCE General Council in terms of student needs and perspectives.

Strongly Agree Agree Neither Agree nor Disagree Disagree Strongly Disagree

****

Positive comments about your experience of having students on ECCE General Council.

Areas for Improvement needed based on your experience of having students on ECCE General Council.
