# Supplementary material for: Is there any benefit to adding students to the European council on chiropractic education evaluation teams and general council? An audit of stakeholders
Source: Chiropr Man Therap. 2019 Oct 13;27:53. doi: 10.1186/s12998-019-0274-7 (PMC6790241; doi:10.1186/s12998-019-0274-7)
Supplement: Supplementary file 2 — Evaluation team member feedback on the use of students on ECCE site evaluation teams. Survey. (PDF 120 kb) [file 12998_2019_274_MOESM2_ESM.pdf]

Additional File 2 (PDF): *Evaluation Team Member* FEEDBACK ON THE USE OF STUDENTS ON ECCE SITE EVALUATION TEAMS

The ECCE has used 1 student on each of the evaluation teams since 2012. Students are to be considered as equal members of these teams and treated accordingly. After several years of experience using students on evaluation teams, the ECCE would like your feedback on your experiences and perceptions of the use of students provide on ECCE evaluation teams.

Please read through each of the 6 questions and select 1 of the 5 answer options to show your level of agreement with each statement. There are 2 further questions at the end where you can record any comments. Thanks so much for your feedback.

1. The student members of the evaluation teams appeared to be treated as equal team members by the other non-student team members.

Strongly Agree   Agree   Neither Agree nor Disagree   Disagree   Strongly Disagree

****

2. Student team members were well prepared for the evaluation.

Strongly Agree   Agree   Neither Agree nor Disagree   Disagree   Strongly Disagree

****

3. Student team members behaved professionally at all times.

Strongly Agree   Agree   Neither Agree nor Disagree   Disagree   Strongly Disagree

****

4. Student team members asked appropriate questions during meetings with institutional representatives.

Strongly Agree   Agree   Neither Agree nor Disagree   Disagree   Strongly Disagree

****

5. Student team members made a unique contribution to the evaluation team.

Strongly Agree   Agree   Neither Agree nor Disagree   Disagree   Strongly Disagree

****

6. Student team members were treated as equal to the non-student team members by individuals at the institutions.

Strongly Agree Agree Neither Agree nor Disagree Disagree Strongly Disagree

****

Positive comments about your experience having students on ECCE evaluation teams:

Areas for Improvement based on your experience having students on ECCE evaluation teams.
