# Supplementary material for: Is there any benefit to adding students to the European council on chiropractic education evaluation teams and general council? An audit of stakeholders
Source: Chiropr Man Therap. 2019 Oct 13;27:53. doi: 10.1186/s12998-019-0274-7 (PMC6790241; doi:10.1186/s12998-019-0274-7)
Supplement: Supplementary file 4 — Student members of ECCE evaluation team: Feedback on the use of students on ECCE evaluation teams. Survey. (PDF 121 kb) [file 12998_2019_274_MOESM4_ESM.pdf]

Additional File 4 (PDF): *STUDENT MEMBERS OF ECCE EVALUATION TEAM:* FEEDBACK ON THE USE OF STUDENTS ON ECCE SITE EVALUATION TEAMS

The ECCE has used 1 student on each of the accreditation evaluation teams since 2012. ECCE states that Students are to be considered as equal members of these teams and treated accordingly. After several years of experience using students on evaluation teams, the ECCE would like your feedback as past student members of evaluation teams on your experiences and perceptions of your contributions, treatment and usefulness to the ECCE evaluation teams.

Please read through each of the 6 questions and select 1 of the 5 answer options to show your level of agreement with each statement. There are 2 further questions at the end where you can record any comments. Thanks so much for your feedback.

1. As a student member of an evaluation team I was treated as an equal team member by the other non-student team members.

Strongly Agree Agree Neither Agree nor Disagree Disagree Strongly Disagree

****

2. I was well informed about my duties as a student member of the team prior to the site visit.

Strongly Agree Agree Neither Agree nor Disagree Disagree Strongly Disagree

****

3. I felt that as a student member of the evaluation team that I had the opportunity to gather relevant information and contribute to the report in a full and meaningful manner.

Strongly Agree Agree Neither Agree nor Disagree Disagree Strongly Disagree

****

4. I felt that as a student member of the evaluation team that I made unique contributions to the evaluation process.

Strongly Agree Agree Neither Agree nor Disagree Disagree Strongly Disagree

****

5. I felt that I was treated with respect and as an equal member of the evaluation team by the institution that was being evaluated.

Strongly Agree Agree Neither Agree nor Disagree Disagree Strongly Disagree

****

Overall the strong points of my experiences as a student member of the evaluation team were:

What unique contributions did you, as a student, make to the team and the evaluation process?
