# Supplementary material for: Is there any benefit to adding students to the European council on chiropractic education evaluation teams and general council? An audit of stakeholders
Source: Chiropr Man Therap. 2019 Oct 13;27:53. doi: 10.1186/s12998-019-0274-7 (PMC6790241; doi:10.1186/s12998-019-0274-7)
Supplement: Supplementary file 5 — All Written Comments to Qualitative Questions Regarding the Students on General Council and Evaluation Teams. (PDF 180 kb) [file 12998_2019_274_MOESM5_ESM.pdf]

Additional File 5 (PDF): Complete Transcription of all Comments to the Qualitative Questions Regarding the Use of Students on ECCE General Council and Accreditation Evaluation Teams.

**Theme:**<br>Use of Students on **ECCE General Council**	**Theme:**<br>**Non-Student Evaluation Team Member** Feedback on the Effectiveness of Student members of ECCE Evaluation Teams	**Theme:**<br>**Institution/Programme** feedback on the use of students on accreditation evaluation team.	**Theme:**<br>**Evaluation Team Student** feedback on the effectiveness of their membership on evaluation teams.
Subtheme:<br>**POSITIVE COMMENTS REGARDING STUDENTS ON ECCE GENERAL COUNCIL (Each individual feedback is separated by the thick black line.)**	***Subtheme:***<br>**Positive comments about your experience having students on ECCE evaluation teams: (individual comments are separated by a solid line.)**	***Subtheme:***<br>**Positive Comments about your Experience having Students on ECCE evaluation Teams: (each person's comments are separated by a solid black line.)**	***Subtheme:***<br>**Overall the strong points of my experiences as a student member of the evaluation team were: (each student's input is separated by a solid line.)**
Positive thinking<br>Provides balance and a different perspective.<br>Students provide unique student perspectives on decisions made by council.<br>-The involvement of students in the council is positive since all levels of stakeholders would need to be represented.<br>Very nice students.<br>Different view. Fresh inputs.<br>Students are key stakeholders in decisions related to the improvement of chiropractic education. They should be involved at all academic levels.<br>Students can provide their unique perspective – student voice- to proceedings.<br>Energy commendable. Students' viewpoints valuable.<br>Very professional<br>Good to have student perspective as a consumer of education.<br>A different point of view is available.<br>Students have a different perspective so can add valuable insight.-Unique perspective of impact related to institutional decisions.	-Students bring a unique contribution and should be maintained.<br>-Students are able to relate to the Institution's students better than other team members and can get information from students that is difficult for the other team members.<br>-They will "see" other aspects of the institution – the students' side of things. Sometimes even go for informal talks.<br>(-Nothing written)<br>-Student members hold a very unique point of view since they can get closer to the students during the site visit.<br>-Student members can add to the dimension of the student-related matters.<br>-Interesting input that made us reflect on certain things.<br>-Students talk to students getting them to reveal things easier.<br>-(Nothing written)<br>-Good to have a student perspective.<br>-Bring a unique and inquisitive perspective to the team, given their direct involvement in present education processes.<br>-Always bring a fresh and different perspective.<br>-Always gain insight from student at the institution that other team members cannot.<br>-Work hard and are organized.<br>-Really useful to have student on the visit. She highlighted areas for questions that the non-student members had not thought of. The students at the institution opened up far more to a peer than to non-	-Opportunity to create a realistic balance and to provide a 'real-world' view of institutions.<br>-Student team members can be very valuable in specifically chosen areas, but not all areas.<br>-Provided the important perspective of students.<br>-Student representative seemed well inducted into the team.<br>-The student was actively involved in the process.<br>-Students provide the potential for interaction being improved between students.<br>-As students are the recipients of what we are all about, it is absolutely essential to have them present as part of the teams. They bring to the exercise a very important perspective and their input is essential and has been of significant benefit to our institution.<br>-They ask unique questions since they graduate in a period that is closer to the cohorts present in the institution.<br>-They also understand things from a student perspective.<br>-Because of their age, they seem to relate better to the students of the institution.	My input and my thoughts were truly valued by the other team members.<br>Meeting the people on the evaluation team was very formative; the team was very welcoming and supportive of my role as a student member.<br>I felt very fortunate to be able visit another chiropractic college, it widens ones horizon, and I feel it is beneficial to gain understanding on how chiropractic is taught in other colleges.<br>Seeing an institution and the build-up of a chiropractic course from the inside was very valuable to form my own views on what content one should expect, and what facilities that should be in place to create a good foundation for such an education.<br>I was able to see things from a slightly different perspective than the non-student members. Both perspectives being important to get the full picture of such an institution.<br>I feel that a student member fills in the gaps which non-student evaluators do not consider or think about because they have not freshly been in the student role.<br>It is always important to have the view of someone who is currently a student to ensure that the course stays current and modern based on what students expect "today". It is important to stay focused on those who are undertaking the

	students it seemed and I suspect, felt more engaged in the process.<br>-The student member also helped me focus on student experience issues which I would have been less aware of had she not been present. I suspect the team was less technocratic as a result.<br>-The most important aspect was helping the team understand local conditions in South Africa. Having had no experience of the profession or the wider country I was ignorant of several professional and social issues.<br><br>-They were always willing to take advice because they needed more advice and training than they had received before arrival on site.<br>-Most knowledgeable were those who were well into their programmes.<br><br>-They were very good at writing up their ideas and in some cases more critical than the professional members.<br><br>-A useful addition to the evaluation team in relation to the easy rapport they can have with the students at the institution.<br><br>-The students provided a unique insight into the student experience at the institution being evaluated. The use of students should definitely continue in future evaluations.<br><br>I had the opportunity to participate at two evaluations with a student team member. At both events, the student team members acted in a very professional way. The first experience was in 2012, the second in 2017. As far as I can recall, I can identify today some differences between the two experiences with student team members with respect to:<br>- Preparation in general<br>- Participation/constructive input at the visitation, during the meetings of the team and the writing of final report<br>In 2017 I personally perceived an improvement in general of the qualitative and quantitative contribution of the student team member to the team.<br>My conclusion: there is definitively an improvement of the input and contribution by student team members between my first and second experience; active and ongoing preparation of these		education, whilst also keeping in mind and including the experience of those who have been qualified or teaching/administering for many years.<br><br>Overall, I had a great experience being a part of the evaluation team. I was treated very well by my team members and the institution. I can strongly recommend other students with the opportunity to be a part of an evaluation team.<br><br>Being exposed to the different organizational levels of a chiropractic institution and understanding its operations.<br>Learning and sharing with an international accreditation evaluation team and understanding the role of ECCE.<br>Meeting and sharing with students, professors and administrators of a foreign chiropractic college.<br><br>I had the opportunity to see how this kind of evaluation is made and what are the most important points every college needs to have in order to offer and education of quality.<br>After this experience, I got more involved in the academic part of chiropractic and became a teaching assistant for the MCC, which has been a great decision for my future as a chiropractor in so many levels.<br>I was able to take advantage on this chance I was given and used it as a way to get out of my comfort zone and get to learn to be less introverted, talk in front of many people in stressful situations and other qualities I'm going to need as a chiropractor for my own practice.<br>I have always seen this experience as a game changer for me in a very helpful way in which I discovered that I was able to do many things I have never thought I could.<br><br>Overall the strong points of my experiences as a student member of the evaluation team were:<br>Overall a very inspiring experience for which I am very grateful. To get the chance to

	members certainly contributes to this positive development.		look under the hub of an educational institution gave new perspective on my own ongoing education. To witness and participate in the interviews, was great inspiration for my own future role/position as a professional.<br>From a student team member point of view: The strong points of being a team member were that I was treated with respect and included fully in all discussions, throughout the evaluation processes by the other team members. The other team members were happy to guide me through the process and advise/inform me where necessary. I was made to feel a valid and equal member with something meaningful to contribute.<br>From an educational point of view: The strong points of my experience were to more fully understand the process of ECCE evaluation of chiropractic programs. This helped me to communicate, to other students the importance of ECCE and what it really means to be evaluated (in the context of educational evaluation). It also allowed me to engage more fully/appropriately within the college management structure, as a member of the Students Union.<br>From a student point of view: The strong points of my experience were to see how another educational establishment delivered student education. There were, of course, large areas of overlap between the education that I was receiving, and the education that they were receiving. However, there were many small areas that demonstrated significant differences in education/structure. Being able to see first hand what the similarities and differences were allowed me to appreciate the scope of education possible and to see that it was necessary for different educational institutions to direct resources in a manner that suited their

			unique situation (physical structures, financial resources, available expertise, management structure, etc.).<br><br>I gained a lot of respect for the institution being a part of the evaluation team, seeing what goes into the selection process for Accreditation.<br>Being able to speak to the other members from other institutions gave me insight into how they may be similar or different to my own.<br><br>Getting the opportunity to analyse all the aspects of a good education program, and hopefully be a tool for its improvement.<br><br>I felt I had a unique understanding of student issues, perceptions of the ECCE process and the importance thereof, the need to involve students as stake holders in their institutions accreditation process.<br>The team were phenomenal in their execution of all aspects of the accreditation visit. I felt that I was made to feel as a valued member of the team and that my input was regarded with equal measure to those of the professional team members.<br>I felt that the students appreciated my presence on the accreditation team and used my presence to their full advantage, in a positive manner.<br><br>I felt I added a unique outlook on the accreditation process and provided a softer contact point for the students whom I interacted with.<br>I felt that I was able to convey the messages of the students to the accreditation team with a unique understanding of those messages.<br>I felt that I asked questions of the accreditation team that had a unique quality to them – given my standing as a student.
Subtheme:	***Subtheme:***	***Subtheme:***	***Subtheme:***

**Areas for Improvement needed based on your experience of having students on ECCE Council. (Each individual feedback is separated by the thick black line.)**	**Areas for Improvement based on your experience having students on ECCE evaluation teams. (Each individual feedback is separated by the thick black line.)**	**Areas for Improvement based on your Experience having students on ECCE Evaluation Teams> (each person's comments are separated by a solid black line.)**	**What unique contributions did you, as a student make to the team and the evaluation process?**
It is a waste of time and money as the students have no clue what is going on and graduate just when they have a bit of experience.<br><br>They need intensive training prior to their 1st meeting along with clear instructions as to their duties and responsibilities.<br><br>ECCE needs to expand the definition of 'student' to include post-graduate training so that students are on council more than 1 or 2 years.<br>―――<br>Engage students more on relevant topics.<br>―――<br>Students need to be better prepared on the role they take in the council, and get more acquainted with the information being displayed.<br>―――<br>Unsure of their role in the council. Unsure if they are able to contribute to the diversity of educational problems.<br>―――<br>Should keep them for a longer time period.<br>―――<br>Assure that students receive proper training and that they have a voice on the council (not simply be a presence, but an actual stakeholder.)<br>―――<br>Training and introduction to ECCE council meetings need to be intensified for students attending, especially for 1st attendees. This will lead to improved and more informed participation and input at meetings.<br>―――<br>Dedication to working with ECCE lacking.<br>Attending meetings lacking.<br>―――<br>I'm not sure the students are well prepared.<br>Students seem to have little input to meetings I have attended.<br>―――<br>Longer period of service for impact and continuity.<br>―――	-None<br>―――<br>-None<br>―――<br>- I think one could have a Skype meeting in advance (1 month – 2 weeks) before the site visit to talk together and divide areas of responsibility. In that way, each member, including students, could deep-dive into the specifics. The pre-visit meeting (on site) could then be used to share and discuss each member's ideas so that the process is both adequate and reflective of the group's concerns.<br>―――<br>-None<br>―――<br>-Student members must always be aware that they need to avoid comparing their institution to the one being assessed. Their experience as a student is valid, but in an evaluation, the main point is to verify the Standards and if the institution fulfills the Standards.<br>―――<br>-I suggest using students in student-related matters.<br>―――<br>-None<br>―――<br>-None<br>―――<br>-None<br>―――<br>-Previous training.<br>-Previous reports need to be stream-lined and compiled so as to have a skeleton form.<br>―――<br>-Some training/induction so they know what to expect prior to the visit.<br>-They can be quite nervous or perhaps feel intimidated by being the only student member of the team.<br>-Training could be online/Skype and quite short in time so not too onerous.<br>―――<br>-The student team member occasionally seemed to lack the confidence to ask probing questions of senior members of staff, (although she did share concerns with the team leader subsequently). Ensuring the student member of the team is empowered to probe by eg helping them appreciate that there is no such thing as a dumb question might help.<br>―――<br>-Give them greater freedom when meeting fellow students because I suspect that some were a bit shy	-Student voice needs to be given equal weight.<br>―――<br>-It is important to carefully consider which areas of the accreditation process students can effectively participate and contribute.<br>―――<br>-Better induct the student.<br>-Encourage the students and support them in asking question from student perspective.<br>-The Chair of the site visit team might emphasize and occasionally repeat the information related to the role of the student as a member of the site visit team.<br>―――<br>-Student evaluators need to perhaps be sensitized to their frame of reference when asking questions and reporting.<br>―――<br>-Try to recruit students early into the process in order to provide some continuity **and** therefore improved contributions throughout the period of time they will be panel members.<br>-Aim for students who are in their 2nd year of education rather than towards the end of their time at the institution whenever possible.<br>―――<br>-I don't have any particular comment at this time that would be relevant to report here.<br>―――	Being a chiropractic student means that you experience firsthand how a college functions, the lectures, student representation, how a student clinic is run etc. This knowledge is useful when discussions during the evaluation process arise, completing the expertise of the other team members. Chiropractic colleges are accredited to guarantee excellence in tuitions for its students. It brings forward the importance of having a student member as part of the evaluation process.<br>―――<br>Being a student whilst on the evaluation team, gave me the benefit of seeing the institution's curriculum, teaching facilities, ethics and goals from a student's perspective. I experienced that the non-students of the team did not necessarily manage to see the curriculum or facilities from a student's perspective, and a balance of perspective between the receiving and giving part of education is quite important to paint a broad picture of it all. This allowed me to contrast and compare the institution to my own university and what is offered there. I think a student who has already completed 3-4 years, as opposed to 1 year like me, would have been able to contribute with even sharper senses, thoughts and ideas in this process. I do not believe this has anything to do with the maturity of the student, but purely their amount of experience and reflections from their own institution. From my experience I found that thoughts on the quality and content of such a degree changed and developed a lot during my 5 years in university, and I think I would have had much more to contribute with towards the end of my course rather than in the beginning.<br>―――

Encourage students to speak up directly addressing them.<br><br>Need to be mature students or post-chiropractic education and specializing.<br><br>Students are probably not knowledgeable in the area of medical education and standards of education plus have little spare time to read the documentation.<br>They can only stay as a member of council for 1 year or less as they graduate.	because accompanied by a professional team member.<br>-Possibly let the Chair have a separate session ahead of the evening meeting before the visit. Confidence and expectations need to be outlined more fully.<br><br>-None<br><br>-Students were less prepared for the evaluation visit than the other team members. This may have since been addressed with revised training methods.<br><br>You are asking for areas of improvement. Honestly, my memory does not allow me today to adventure myself on a slippery ground by developing precise comments on specific areas.		In my opinion, the student member has an important role in the accreditation process. It represents the voice of the students in the best way possible. Having your own education´s everyday life fresh in mind, makes you able to put yourself in the place of other students. This contributes with other angles on the accreditation process and gives a good understanding of the students there. Finally, students as a part of the evaluation team is an ideal way to exchange ideas and experiences between institutions from the perspective of the students.<br><br>Thorough communication with the students of the institution that was being evaluated.<br>Student viewpoint on organization of the institution and student welfare.<br>Comparing the different areas evaluated with the institution where I was studying that was already accredited.<br>Sharing an innovative and contemporary viewpoint on chiropractic education with the evaluation team.<br><br>As a student, I think my most important contribution was the fact that I was the only member who could talk Spanish properly, which was very helpful not only to find good places to have dinner, but in order to understand members of the staff who could not express themselves and also I was able to translate all the documents which were being given in the clinic which were in Spanish.<br>Also, I could appreciate some things as a student which were not appreciated by the rest of the staff, such as benefits or absences of students.<br><br>To the team: As a team member I felt respected and listened to as an equal. I supplied the team with my unbiased, honest opinion and view.<br>To the evaluation process: I focused on certain areas. I spent time assessing physical facilities, talking with students and observing the student clinic facility-but also weighing

			in on all other Standards in the evaluation process.
			Each of the other members of the team has been a student, at university level, in the past and can therefore draw on their experience of the issues that may be important to students. So, as a student at the time of the evaluation, I am not convinced that I provided a specifically 'unique' contribution.<br>What I do think I contributed as a student team member, is perhaps a more immersed perspective of the current issues that student are, or may be, facing. This allowed me to identify with students, perhaps more easily than the other ream members, and to view the program structure and management more directly, from the perspective of how it could affect me as a student and future practitioner.
			I felt being from the same country as the institution being evaluated it allowed insight in certain aspects; for example, when it came to making the clinic paperless, how with electricity in our country it may not be possible. Some of the other members of the team may not have understood the circumstances.<br>Being a final year student at the time it was also easier to understand the work pressure that students may have, having been through the program at my own institution.<br>Being able to contribute in ways to improve certain aspects or faults that may have been picked up.
			It was long ago but I think that as a student at that time, I connected with the students and tried to transmit their discomforts and desires.
